# Squamous cell carcinoma of the rectum: a consequence of immunosuppression resulting from inhibiting tumour necrosis factor (TNF)?

**DOI:** 10.3332/ecancer.2016.646

**Published:** 2016-06-07

**Authors:** Alexandra Silverton, Roy A Raad, Leah Katz, Andrea Downey, Franco M Muggia

**Affiliations:** 1 Department of Medicine, Perlmutter Cancer Center at the New York University Langone Medical Center, New York, NY 10016, USA; 2 Department of Radiology, Perlmutter Cancer Center at the New York University Langone Medical Center, New York, NY 10016, USA; 3 Department of Radiation Oncology, Perlmutter Cancer Center at the New York University Langone Medical Center, New York, NY 10016, USA

**Keywords:** TNF antagonists, squamous cell cancer, rectal cancer, secondary malignancies, rheumatoid arthritis, etanercept

## Abstract

Treatment with tumour necrosis factor (TNF) antagonists may lead to enhanced susceptibility to certain malignancies. In particular, an association is seen emerging between TNF antagonists and development of squamous cell carcinomas (SCCs) of the skin (in association with psoriasis), the oral cavity, and in the anogenital areas (possibly related to prior human papilloma virus infection). We present here a case of a 53-year old woman with a history of severe rheumatoid arthritis (RA), most recently treated with the TNF antagonist etanercept plus methotrexate, presented to our service after several months of increasing left pelvis and buttock pain. Evaluation with a computerised tomography (CT)-directed biopsy of a pelvic side wall mass revealed a metastatic SCC. On a fluorodeoxyglucose (FDG) positron-emission tomography (PET) an additional area of uptake was identified in the left posterior rectum corresponding to a 1 cm nodule palpable on digital exam. Colonoscopic biopsy revealed a basaloid SCC of the rectum as the likely primary site. Immunosuppression following TNF antagonist therapy may have given arise to this unrestrained neoplastic growth. It thereby underscores the need for an initial baseline study of risk factors and identification of patients who are at higher risk for development of a malignancy, in order to achieve a diagnosis at an early stage.

## Introduction

Tumour necrosis factor (TNF) is a pro-inflammatory cytokine that plays an important role in immune response and is implicated in the pathogenesis of many inflammatory conditions [1–4]. Etanercept is a genetically engineered soluble fusion protein that consists of the TNF-receptor p75 receptor fused to the Fc portion of human immunoglobulin G1 (IgG1) which binds and inactivates TNF. It first gained FDA approval in 1998 and is now used for treatment of psoriasis, ankylosing spondylitis, and in moderate-to-severe rheumatoid arthritis (RA). Studies have demonstrated that treatment with etanercept results in significant clinical benefit with minimal associated toxicity in patients with RA who have had inadequate responses to other disease-modifying drugs [4–6]. We report here a patient with severe RA who was diagnosed with an unusual rectal squamous cell carcinoma (SCC) while on treatment with etanercept. Primary squamous cell rectal cell cancer is an extremely rare entity which constitutes only 0.1–0.25% of all colonic cancers. It more commonly affects women than men and typically presents in the fifth and sixth decade of life. Given its rarity, optimal treatment is not well delineated. In the past, the majority of patients were treated with surgery similar to rectal adenocarcinoma but newer studies suggest that primary chemo-radiotherapy may be an acceptable alternative to surgery [7, 8]. Pathogenesis is also unclear although it has been suggested that human papilloma virus (HPV) type 16 and 18 may play a role in a similar manner to cervical, head and neck, and anal cancer [9]. In a study by Sotlar *et al*, molecular biologic analysis using polymerase chain reaction (PCR) and *in situ* hybridisation demonstrated the presence of HPV-16 as well as transcriptional activity of the viral E6/E7 oncogenes in specimens from the rectal mucosa of a patient with rectal SCC suggesting HPV as a possible aetiologic agent. However, some reports suggest there is no obvious link between HPV and squamous cell carcinoma of the rectum [10–11]. Study of the pathogenesis of tumours resulting from inhibiting TNF may provide further insight into the role of this cytokine in restraining the growth of SCC of various origins.

## Case report

A 53-year old woman with severe rheumatoid arthritis (RA) presented to her primary care physician in March 2014 with increasing left pelvis and buttock pain over several months. Treatment for her RA dated back to 1995 and included several regimens including steroids, methotrexate alone, and for the past six years she appeared to have benefitted from the addition of etanercept to methotrexate (50 mg weekly). In addition to the pelvic pain, she was experiencing fatigue but there were no neurologic signs, no weight loss, fevers, or night sweats. A computed tomography (CT) scan of the abdomen and pelvis revealed a lobular, infiltrative 3.1 x 6.1 x 4.4 cm soft tissue mass extending along left obturator space with encasement of the inferior branch of left internal iliac vessels and likely involvement of the sciatic bundle with extension to the left vaginal wall.

**Figure 1a–c.**
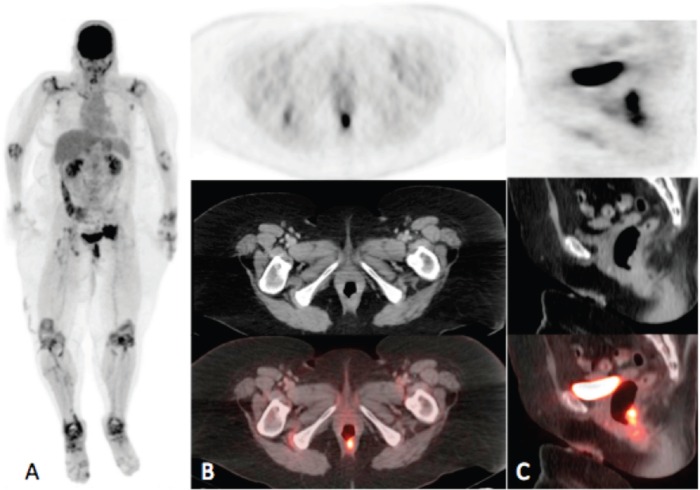
Maximum intensity projection (MIP) (A) and PET, CT and fusion images in the axial (B) and sagittal (C) planes. MIP image demonstrates intense uptake in the rectum and in the pelvis to the left of the urinary bladder. Axial and sagittal images demonstrate intense uptake (SUVmax = 17.4) localising to a rectal soft tissue mass. Note on the MIP image symmetric bilateral circumferential hyper-metabolism surrounding the joints in the upper and lower extremities consistent with the known history of rheumatoid arthritis.

**Figure 2a–c.**
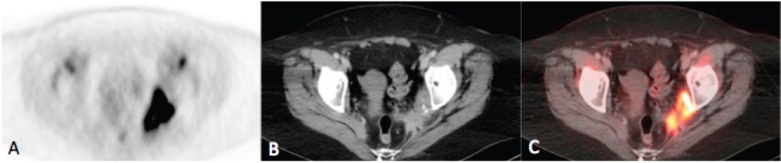
Axial PET(A), CT (B) and fusion (C) images of the pelvis demonstrating intense uptake (SUVmax = 21.9) within an irregularly marginated soft tissue mass at the pelvic sidewall containing punctate calcifications, consistent with the biopsy-proven metastatic squamous cell carcinoma.

CT-guided core biopsy was consistent with squamous cell carcinoma (SCC), poorly differentiated. A fluorodeoxyglucose (FDG) positron-emission/computerised tomography (PET/CT) scan demonstrated a markedly FDG-avid left femoral lymph node and intense uptake in the left posterior rectum corresponding to a 1 cm nodule palpable on digital exam that was concerning for malignancy (Figure 1a–c) in addition to the previously identified left pelvic mass (Figure 2a–c) that was responsible for her initial symptoms. Further workup with colonoscopy and biopsies confirmed an unusual basaloid SCC of the rectum approximately 5 cm above the anal verge with positive p16 expression, a surrogate marker for HPV status. After multidisciplinary discussion we concluded that the picture was consistent with an unusual rectal primary malignancy with left pelvic and femoral nodal metastases. While controlling her RA with prednisone alone, she was started on treatment with chemotherapy (oxaliplatin and capecitabine) and concurrent radiation therapy.

Radiation consisted of treatment to the pelvis with a 3D-conformal four-field set-up with a dose of 4320 cGy in 24 fractions using a mixed 16/6 MV photon beam. Treatment to the pelvis was sequentially followed by a boost to the pelvic mass to a dose of 1000 cGy in five fractions using a 3D-conformal anterior-posterior field set-up with a 16 MV photon beam. The patient experienced a gradual response with eventual full relief of symptoms. At completion, she relocated her care to Florida where she was consolidated with seven cycles of carboplatin and paclitaxel. She was followed until September 2015 but details of her disease status are unknown.

## Discussion

The association of TNF antagonists and development of SCCs has come to light in several reports [12–21]. Patients with psoriasis exposed to therapeutic regimens based on ultraviolet light were found to be at an increased risk of cutaneous SCC(cSCC) if treated with TNF antagonists [13, 15, 16, 22]. A role of TNF in promoting programmed cell death of tumour cells through CD8+ T-cell induction and natural killer cell stimulation may be implicated in potentiation of tumour growth after TNF inhibition [23]. Potential confounders in the association between risk of malignancy and TNF antagonists may be that the underlying conditions for which TNF antagonists are being given are themselves associated with higher rates of malignancies [22, 24, 25].

In fact, one such study of the Swedish inpatient registry compared 53,067 RA patients admitted to hospital between 1990–2003 to patients treated with TNF antagonists for RA during 1999–2003 (n = 4160). Results demonstrated an increased occurrence of non-melanoma skin cancers (standardised incidence ratio = 3.6) but no rise in solid tumours frequency in the TNF antagonist-treated group. In fact, patients treated with TNF antagonists actually had a 20% decreased risk for breast cancer that echoed those of the inpatient registry as well as historical RA cohorts. However, the comparator RA cohort did have a 25% reduction in colorectal cancer relative to other admissions that was not seen in the TNF antagonist treated patients [12]. Patients with RA were at increased risk of lymphoma and leukaemia with standardised incidence ratios of 2.0 and 2.2, respectively. Although patients in this study treated with TNF antagonists had a three-fold higher risk of lymphoma than the general population, when adjusted for age, sex, and disease duration, but this risk for lymphoma was no higher than in other RA cohorts [25]. A subsequent meta-analysis by Bongartz *et al* of nine randomised placebo-controlled trials of TNF antagonists in RA, found a malignancy of 0.2% in controls who received only placebo versus 0.8% in patients receiving anti-TNF medications for their RA. The number needed to harm was 154 (95% CI, 91–500) for one additional malignancy within a treatment period of 6–12 months [26]. These large studies point to the complexity of defining an increase in the development of malignancy in patients with RA who are also receiving anti-TNF therapies.

A case report by Smith and Skelton (2001) described seven patients with RA who experienced one or more rapidly growing cSCC that developed shortly after the initiation of etanercept therapy [19]. Similar results were found in a 2011 discussion of four patients with psoriasis started on etanercept who developed cSCC within a relatively short interval after starting treatment [13]. The average time of SCC onset after beginning treatment was 11 months (range 1–17 months). Another report in 2007 described a case where a man developed multiple sCC and keratoacanthomas within ten weeks of starting etanercept for treatment of psoriasis [17]. After treatment was stopped, there was regression of the keratoacanthomas within a few weeks. Two cases reported rapid onset of cSCC of the penis in patients receiving etanercept for psoriasis [15, 16].. Interestingly, both of these patients tested negative for HPV. One of these reports had a fatal outcome as a result of metastasis [15].

SCCs of the oral cavity have also been reported with TNF antagonist therapy [14, 18, 21]. Chainanin-Wu *et al* (2014) described a case of a 45-year-old man with RA who was started on etanercept and within two months developed oropharyngeal pain and ulcerations and was diagnosed with tonsillar carcinoma [14]. Biopsy showed poorly differentiated SCC, and immunohistochemistry with expression of p16 in the tumour cells which is a marker of HPV virus. Another report described the development of invasive SCC of the lower lip in a 29-year old patient who had been taking adalimumab for ankylosing spondylitis for two years [21]. Although many of these patients had prior history of tobacco or alcohol use, these cases include instances of oral squamous cell cancers (oSCC) after treatment with TNF antagonists in the absence of the usual risk factors for oropharyngeal cancer [18].

## Conclusions

In conclusion, our report of the rare occurrence of a basaloid SCC of the rectum with positive p16 expression occurring after treatment of RA arthritis with etanercept and methotrexate also raises questions about enhanced susceptibility to human papilloma virus (HPV) as a risk factor for secondary malignancies following TNF inhibitor use. Of possible relevance, TNF antagonists have been noted to exacerbate genital HPV recurrences [28]. Therefore, further research to characterise the effect of TNF antagonists on control of HPV and their possible role in secondary malignancies following treatment is needed. The relationship between immunosuppression and unleashing malignant growth suggests a possible therapeutic role for immune checkpoint inhibitors when faced with advanced squamous malignancies, including those arising in the rectum that may have resulted as a consequence of HPV infection. There are several ongoing clinical trials that are attempting to answer this question for SCCs of the head and neck as well as the oesophagus [29–34]. Until such data come to light, patients who are initiating treatment with TNF antagonists should receive counseling on sun protection, education about regular self examination and dermatologic exams, and age-appropriate preventive screening measures such as colonoscopy. Increasing awareness about the potential risks of TNF antagonist therapy should facilitate not only early diagnosis but an initial baseline study of risk factors to determine which patients might be at higher risk.

## Conflicts of interest

The authors have no conflicts of interest to declare.
